# MRI findings are often missed in the diagnosis of Creutzfeldt-Jakob disease

**DOI:** 10.1186/1471-2377-12-153

**Published:** 2012-12-05

**Authors:** Christopher Carswell, Andrew Thompson, Ana Lukic, John Stevens, Peter Rudge, Simon Mead, John Collinge, Harpreet Hyare

**Affiliations:** 1 MRC Prion Unit and Department of Neurodegenerative Disease, UCL Institute of Neurology, and National Prion Clinic, University College London Hospitals NHS Trust, WC1N 3BG, London, UK; 2 Department of Neuroradiology, National Hospital for Neurology and Neurosurgery, University College London Hospitals NHS Trust, WC1N 3BG, London, UK

**Keywords:** Prion, MR imaging, Creutzfeldt-Jakob disease, Dementia

## Abstract

**Background:**

Establishing a confident clinical diagnosis before an advanced stage of illness can be difficult in Creutzfeldt-Jakob disease (CJD) but unlike common causes of dementia, prion diseases can often be diagnosed by identifying characteristic MRI signal changes. However, it is not known how often CJD-associated MRI changes are identified at the initial imaging report, whether the most sensitive sequences are used, and what impact MRI-diagnosis has on prompt referral to clinical trial-like studies.

**Methods:**

We reviewed the MRI scans of 103 patients with CJD referred to the National Prion Clinic since 2007 and reviewed the presence of CJD-associated changes, compared these findings with the formal report from the referring centre and reviewed the types of sequence performed.

**Results:**

In sCJD we found CJD-associated MRI changes in 83 of 91 cases (91% sensitivity). However, the referring centres documented CJD-associated MRI changes in 43 of the sCJD cases (47% sensitivity). The most common region not documented by referring centres was the cortex (23 of 68 sCJD cases), but there was a statistically significant discrepancy in all regions (p<0.0001). Patients in whom MRI abnormalities were missed by the referring hospital were more advanced at the time of recruitment to a clinical trial-like study (p=0.03).

**Conclusions:**

CJD-associated MRI changes are often not documented on the formal investigation report at the referring centre. This is important as delay makes enrolment to clinical trials futile because of highly advanced disease. If a diagnosis of CJD is suspected, even if the initial imaging is reported as normal, a specialist MRI review either by an experienced neuroradiologist or by a prion disease specialist unit could facilitate earlier diagnosis.

## Background

Creutzfeldt-Jakob disease occurs as a sporadic, iatrogenic and genetic fatal neurodegenerative disorder [1]. With the exception of genetic types, CJD can only be definitively diagnosed by tissue examination, usually brain biopsy or at autopsy. However, using clinical and investigative criteria, WHO sought to define the likelihood that a patient has CJD. The original criteria include dementia with variety of neurological signs, the presence of 14.3.3 proteins in the CSF and characteristic EEG with pseudo-periodic complexes. More recently MRI criteria have been added giving a sensitivity and specificity >90% for probable CJD [2,3]. These changes comprise alterations on diffusion weighted (DWI) and FLAIR images in the basal ganglia, thalamus and cortex [4,5]. However these changes can be subtle and easily missed by a clinician unfamiliar with such a rare disorder. With the development of potential therapies to alter the natural history of the disease early diagnosis is essential to give the maximum chance of salvaging functional brain tissue [6]. As MRI is the most widely available non-invasive test assisting diagnosis, it is important that clinicians are aware of the imaging features. At present it is not known how often the MRI abnormalities are reported by radiologists in cases of definite CJD. We therefore reviewed images from 133 consecutive patients referred to the National Prion Clinic and compared the report from the referring clinician and our own specialist radiologists; 103 were suitable for the study of whom 70% had an autopsy or cerebral biopsy.

## Methods

### Experimental procedures

#### Patients

In 2004 the Chief Medical Officer wrote to UK neurologists requesting that all patients with suspected CJD are referred to both the National CJD Surveillance Unit in Edinburgh and the National Prion Clinic (NPC) based at the National Hospital for Neurology and Neurosurgery (NHNN), London. All patients underwent PRNP analysis for genetic mutations. MRI scans from all visited patients are requested by the NPC. The scans studied in this work were from patients who were referred to the NPC by their local physician with full consent and the patients were enrolled into either the clinical trial PRION-1 or the on-going National Prion Monitoring Cohort study. Both studies were approved by the Eastern Medical Research Ethics Committee and are compliant with the Helsinki Declaration.

#### MRI evaluation and reporting

##### NPC MRI review

The first MRI scan were reviewed by one of two senior neuroradiologists (H.H. and J.S.) at The National Hospital for Neurology and Neurosurgery (NHNN and the NPC). The radiologists were aware of the suspicion of CJD at the time of reporting but were not aware of the results of any biopsy or post mortem results at the time of reporting. The available MRI sequences were reviewed together as the sensitivity of individual sequences was *not* being compared. Three types of high signal intensity lesions were accepted as CJD-associated MRI changes on DWI and/or Fluid Attenuated Inversion Recovery (FLAIR) and/or T2-weighted sequences:

1. Lesions in the striatum (caudate or putamen or both)

2. Lesions in the thalamus including the pulvinar

3. Lesions along the cortical ribbon (cerebral or cerebellar)

##### External MRI formal report review

All external reports were reviewed for whether:

1. The MRI was normal

2. The MRI differential diagnosis included CJD

3. CJD-associated abnormalities were specifically documented in the caudate, putamen, thalamus or cortex

#### Exclusion criteria and classification

All patients in whom a clinical diagnosis other than CJD was suspected, or those who did not fulfil the criteria for CJD were excluded, as were all scans before 2007 when specific scan review was not routine. Patients who were referred to the NPC with a suspected diagnosis of inherited prion disease (IPD) were also excluded, as prion gene sequencing resulted in a definite diagnosis and MRI rarely shows any signal change with the majority of mutations. Two cases with the E200K mutation and one case with the P102L mutation detected at a later stage were included as the clinical picture at the time was indistinguishable from sCJD.

The remaining patients were categorised by CJD type with all available investigations; those with sporadic CJD were further sub-categorised at the time of initial referral as: possible, probable or definite using the WHO criteria. We then re-classified the sCJD patients using the modified criteria suggested by Zerr et al. [3] for both the referred MRI report and then our own MRI impression to see whether our MRI interpretation altered the classification of these patients. Where available, the scales data was documented, based on a composite functional scale derived from the Barthel ADL index and Clinical Dementia Rating Scale Sum of Boxes.

### Statistical analysis

The patient population characteristics and the comparison of the presence of CJD-associated MRI images between NPC and referring centre were assessed using the Chi2 test (GraphPad Prism).

### Results

We obtained 133 MRI scans from 113 patients referred to the NPC between 01.03.2007 and 24.05.2010 with a suspected diagnosis of CJD. 30 scans were excluded: 21 cases did not fulfil the WHO diagnostic criteria for CJD or were thought clinically to be due to a different cause; the referring report could not be obtained in 9 cases.

A total of 103 reported scans were available for review of which 56% were from males. The eventual diagnosis was sCJD in 91 cases with a median age of onset of 65 years (3 possible, 35 probable and 53 definite). Two cases of iatrogenic CJD (iCJD) from cadaver sourced growth hormone were identified (both 41 years old when symptomatic), 3 patients with inherited prion disease and 7 patients had vCJD with a median age of onset of 22 (2 probable and 5 definite) (Table 1).

**Table 1:** The characteristics and MRI report comparison of 103 CJD patients

	**Final diagnosis (n)**				**Median age of Onset (min-max)**	**No. DWI sequences (%)**	**No. cases with “CJD-associated changes” on MRI (%)**		**No. cases from neurological centre (%)**	**No. cases with unreported changes from neurological centre (%)**
	**Possible**	**Probable**	**Definite**	**Total**			**NPC review**	**Referring centre**		
**sCJD**	3	35	53	91	65 (38–85)	75 (82)	83 (91)	43** (47)	55 (60)	19 (43%)
**vCJD**		2	5	7	22 (20–53)	6 (86)	7* (100)	7 (100)	5 (71)	0
**iCJD**		2		2	41 (0)	1	2	0	2 (100)	2 (100)
**IPD**			3	3		1			-	-
E200K			2		67 (63–70)	-	2	0	0	-
P102L			1		61 (0)	-	1	1	0	-

The scans and corresponding reports of 103 CJD patients were reviewed. The population comprised 91 sCJD, 7 vCJD, 2 iCJD and 3 IPD patients. The number of scans with DWI is shown as is the incidence of CJD-associated changes as noted by NPC and referring centre.

*= 2 of the 7 patients had MRI changes normally associated with sCJD.

**= p<0.0001 compared with NPC opinion of CJD-associated changes using Chi^2^ with 1df and Yates’ correction.

In sCJD, we found characteristic increases in MRI signal intensity in 83 of the 91 cases at NPC review, a sensitivity of 91% for MRI in the diagnosis of CJD in this series (Table 1). CJD-associated changes were not detected in 8 a total of cases, 2 of whom did not have a DWI (2 definite sCJD). The remaining 6 cases had a DWI sequence performed (two definite, three probable and one possible sCJD) (Table 1). The referring centres, however, described CJD-associated MRI changes in 43 of the 91 sCJD cases (sensitivity of 47%), with unreported CJD-associated MRI changes in 40 of the 83 sCJD cases found to have MRI changes at NPC review (p<0. 0001 compared with our identification of similar changes using Chi^2^ with Yates’ correction and 1 degree of freedom). In cases with unreported CJD-associated MRI changes, there was no significant difference between cases referred from a neurological centre or general hospital (Table 1). Occasionally CJD-associated MRI changes were commented upon but thought to be calcification of the basal ganglia, but more commonly there was no mention of critical thalamic/cortical signal changes.

**Figure 1:**
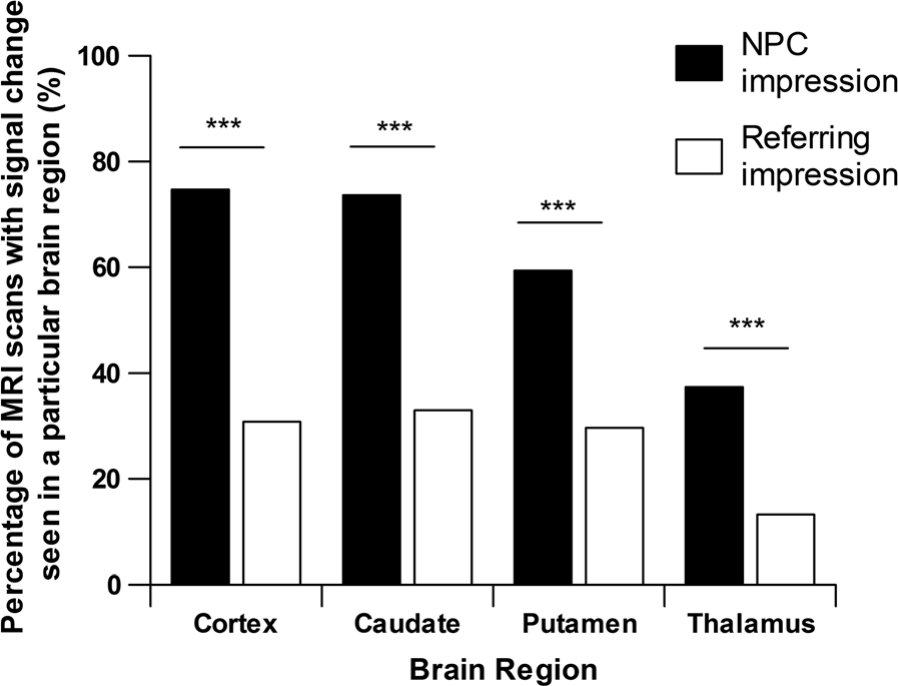
**The MRI scans of 91 patients with sCJD were reviewed and compared with the corresponding report from the referring centre.** The overall sensitivity of MRI scans found by the NPC was 91% compared with 47% from the referring centre (p<0.0001). The NPC found that 74% of cases had cortical high signal, 73% of cases had changes in the caudate, 59% of cases had changes in the putamen and 37% in the thalamus. The region that there was the most discrepancy between the two impressions was the cortex but there was a significant difference in all regions evaluated (p<0.0001).

To further investigate whether referring centres identified CJD-associated MRI changes we reviewed specific locations of increased MRI signal in sCJD cases (Figure 1). We found that the most common sites for CJD-associated MRI changes were cortex (74%) (Additional file 1: Figure S1B and S1C) and caudate (73%) with thalamus being the least affected (37%) (Figure 1). The most common region not documented by referring centres was the cortex (23 of 68 sCJD cases), but there was a statistically significant discrepancy in all regions (p<0.0001 Figure 1).

All reports of vCJD were concordant with our own. Interestingly, two definite cases did not have a pulvinar signal increase greater than that of the basal ganglia and were initially classified as sCJD. In contrast, both iCJD cases were initially reported to have had normal scans (Table 1, Additional file 1: Figure S1A). When the diagnosis was categorised according to the modified criteria suggested by Zerr et al. [3] at the time the patient presented to the NPC, using the MRI report and our own MRI impression, we found that the diagnosis changed from possible to probable sCJD in only two cases as all other patients with negatively reported scans had either characteristic EEG changes or positive CSF 14–3-3 protein (data not shown).

DWI sequences were performed in 83 of the 103 MRI studies (79%) (Table 1). In the 20 patients for whom DWI was not performed, CJD-associated MRI changes were detected in 16 MRI studies. In all the MRI studies, FLAIR and T2-weighted sequences were present.

A subset of the cohort which included 62 patients were rapidly reviewed by the National Prion Clinic team and scales data obtained based on a composite functional scale derived from the Barthel ADL index and Clinical Dementia Rating Scale Sum of Boxes. Where the MRI abnormalities were detected by the referring hospital (n=40), patients had a significantly higher (less advanced disease) (p=0.03) median functional score of 5/35 (range 0–31) compared to median MRC score of 2/35 (range 0–27) when the MRI abnormalities were missed by the referring hospital (n=22).

### Discussion

We have shown that MRI signal changes are often not identified on initial scan at referring hospitals in patients with CJD. Signal abnormality characteristically occurs in the basal ganglia and thalamus [7-9] but this was reported in only about half the referred patients and cortical abnormalities were reported in even fewer patients. Missed diagnosis was associated with a more advanced clinical state at enrolment in clinical-trial like studies.

There are a number of reasons why the MRI signal abnormalities were not reported by the referring clinicians. Firstly, the rarity of CJD means radiologists, even in a neurological centre, have not seen many cases and will be unfamiliar with the characteristic MRI findings. Secondly, it is possible that CJD at the time of original scanning was not suspected, or was not mentioned on the original request for MRI scanning, which would make the radiologist consider a much wider range of possibilities. The heightened suspicion of CJD at referral to our centre is highly likely to be a factor in the increased ascertainment. It is possible that after joint multidisciplinary meetings with added clinical information, new interpretations of the scan were not updated on the actual MRI report.

Thirdly, abnormality can be difficult to detect when the MRI is degraded by movement artefact, a common phenomenon in confused patients with CJD. In these situations, it is even more imperative to perform a DWI sequence which is relatively short compared to other MRI sequences and can provide good quality information in an uncooperative patient. Despite the inclusion of DWI sequences in many routine MRI protocols, we found that DWI was only performed in 79% of MRI studies. When a patient is uncooperative, performing the DWI sequence earlier in an MRI protocol may be more helpful than persisting in acquiring good quality images in other sequences.

Finally, the MRI abnormalities may have been detected but dismissed as artefact and not reported. Increased signal abnormality in the neocortex was seen in 17% of controls subjects in a study by Young et al. [4] which could lead to caution in interpretation of DWI findings by many radiologists. However, visualisation of the DWI-trace image in conjunction with the Apparent Diffusion Coefficient (ADC) map where corresponding decreased signal is seen (Additional file 1: Figure S1D), can confirm initial suspicion of DWI abnormalities. Performing an additional DWI sequence at a high b-value such as b=3000 s/mm^2^ can increase confidence when DWI signal abnormality is suspected [10]. DWI using a standard b-value of 1000 s/mm^2^ and a high b-value of 3000 s/mm^2^ are routinely performed as part of the MRI Protocol for the diagnosis of prion diseases at our institution. Asymmetrical cortical or deep grey nuclei involvement can also increase confidence in abnormal signal interpretation. Nevertheless cortical ribboning must be interpreted with caution; this particularly applies to use of machines with field strength higher than 1.5 T used in the present study.

We found evidence of a less advanced clinical state once patients were referred to our centre in patients where the MRI abnormalities were detected by the referring centre compared to those where the MRI abnormalities were initially missed. At present a high proportion of CJD patient present to specialist centres with very advanced neurodisability (often within a few days of death) when clinical trials are futile. MRI is usually one of the initial investigations when a patient is admitted which could allow a clinical diagnosis to be made at an earlier stage, avoiding uncertainty amongst patient relatives and the need for further unnecessary investigations. We believe that in patients where the initial MRI findings were missed by the referring centre, further investigation delayed referral leading to a more advanced clinical state at specialist referral.

### Conclusion

MRI is a useful tool in the diagnosis of CJD but the characteristic changes are often not identified at initial scan in the early stages of disease. This does not imply an adverse criticism of the radiologists who have probably never, or rarely seen, an MRI from a patient with CJD and to whom the diagnosis has not been raised as a possibility. However CJD MRI findings have been reported in the literature for over a decade and it is important if experimental therapeutics are to be successfully tested in human trials. If a diagnosis of CJD is suspected, even if the initial imaging is interpreted as normal, specialist MRI review either by an experienced neuroradiologist or by a prion disease specialist unit could facilitate earlier diagnosis.

## Competing interests

The authors declare that they have no competing interests.

## Authors’ contributions

CC, PR, SM, JC and HH designed the study. CC, AT, AL, PR and SM collected data. The raw data was analysed by HH and JS. The data was then cleaned and analysed further by CC, HH, PR and SM. Statistical analysis was performed by CC, HH and SM. Figures were written by CC and HH with review by AT and AL. The paper was initially drafted by CC, PR, SM and HH before internal presentation and analysis by all contributing authors. The final paper was revised by CC, PR, SM, JC and HH. JC is responsible for the overall content as guarantor. All authors read and approved the final manuscript.

## Pre-publication history

The pre-publication history for this paper can be accessed here:

http://www.biomedcentral.com/1471-2377/12/153/prepub

## Supplementary Material

Additional file 1: Figure S1 Selection of 4 cases from the 40 cases where CJD-associated MRI changes were missed at the referring centre. (**A**) A probable iCJD (growth hormone treated) patient: axial T2W images demonstrates hyperintensity within the basal ganglia and thalamus bilaterally; (**B**) A “probable” sCJD patient: axial DWI images show cortical hyperintensity in the frontal cortex bilaterally; (**C**) A “definite” sCJD patient: axial DWI shows hyperintensity in the head of caudate nucleus and occipital cortex bilaterally; (**D**) A “definite” sCJD patient: axial ADC map demonstrates restricted diffusion in the head of caudate and putamen bilaterally. Click here for file
