# Supplementary figures and images for: MRI findings are often missed in the diagnosis of Creutzfeldt-Jakob disease
Source: BMC Neurol. 2012 Dec 5;12:153. doi: 10.1186/1471-2377-12-153 (PMC3604954; doi:10.1186/1471-2377-12-153)

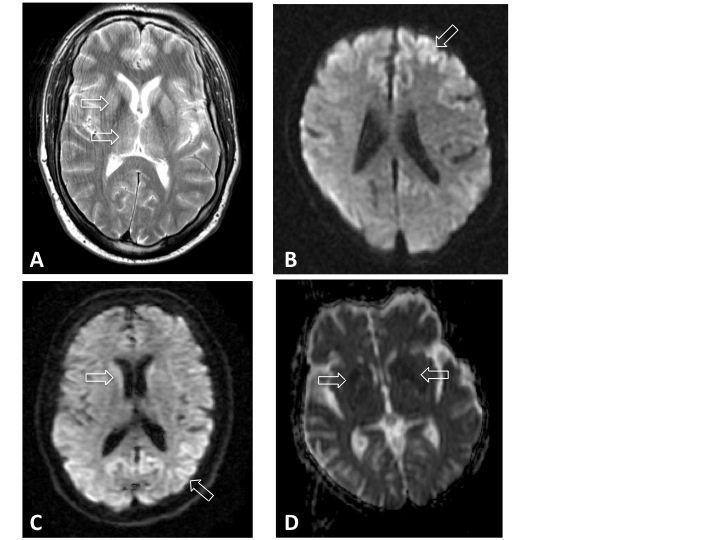

Supplement: Additional file 1: Figure S1 — Selection of 4 cases from the 40 cases where CJD-associated MRI changes were missed at the referring centre. (A) A probable iCJD (growth hormone treated) patient: axial T2W images demonstrates hyperintensity within the basal ganglia and thalamus bilaterally; (B) A “probable” sCJD patient: axial DWI images show cortical hyperintensity in the frontal cortex bilaterally; (C) A “definite” sCJD patient: axial DWI shows hyperintensity in the head of caudate nucleus and occipital cortex bilaterally; (D) A “definite” sCJD patient: axial ADC map demonstrates restricted diffusion in the head of caudate and putamen bilaterally. [file 1471-2377-12-153-S1.tiff]
